# Effect of Hind- and Fore-Foot Eversion on Positional and Rotational Displacement of the Knee in Standing Posture

**DOI:** 10.3390/healthcare11222931

**Published:** 2023-11-09

**Authors:** Jae Yi Kim, So Yeong Park, Do Yeon Lee, Seong Hoon Jeong, Il Soo Kim, Seong Hoon Lim

**Affiliations:** 1 Biomechanics Research and Development Center, Rhin Rehabilitation Hospital, Yongin-si 16864, Republic of Korea; 2 Shinsegae I&C AI Lab, Seoul 04529, Republic of Korea; 3 Department of Rehabilitation Medicine, Seoul St. Mary’s Hospital, College of Medicine, The Catholic University of Korea, Seoul 06591, Republic of Korea; 4 CMC Institute for Basic Medical Science, Catholic Medical Center of The Catholic University of Korea, Seoul 06591, Republic of Korea

**Keywords:** knee osteoarthritis, insole, external knee adduction moment, moment arm, foot eversion

## Abstract

We investigated the effects of hindfoot and forefoot eversion on the knee’s positional and rotational displacement, plantar pressure, and foot discomfort in a standing posture, beyond the traditional focus on external knee adduction moments (EKAM) in lateral wedge insoles. Twenty-six healthy participants underwent hindfoot eversion from 0 to 10 degrees in 2-degree increments, and forefoot eversion from 0 degrees to the hindfoot eversion angle in 2-degree increments in a standing posture. At each eversion angle, the knee’s medial displacement, EKAM’s moment arm decrease, plantar pressure changes, and foot discomfort were obtained and compared across varying angles. Both hindfoot-only and entire-foot eversion led to significant medial knee displacement and the EKAM’s moment arm decrease, with more pronounced effects in entire-foot eversion. At each hindfoot eversion angle, increasing forefoot eversion resulted in significant medial knee displacement and EKAM’s moment arm decrease. Lower leg rotations were not significantly affected in hindfoot-only eversion but displayed significant medial tilting and internal rotation in entire-foot eversion at specific combinations. Varying eversion angles significantly influenced the forefoot pressure, with heel pressure remaining unaffected. Notably, the lateral forefoot pressure increased significantly as the forefoot eversion angle increased, particularly at higher hindfoot eversion angles. Foot discomfort increased significantly with higher eversion angles, particularly in entire-foot eversion, and also increased significantly as the forefoot eversion angle increased at higher hindfoot eversion angles. Insole configurations incorporating 6–10 degrees of hindfoot eversion and 40–60% forefoot eversion of the hindfoot angle may offer optimized biomechanical support for knee osteoarthritis patients.

## 1. Introduction

Knee osteoarthritis (OA), a highly prevalent chronic joint disease imposing a global healthcare burden [1,2], is closely associated with external knee adduction moments (EKAMs), a widely used measure of joint load and a key factor in disease severity [3,4,5]. The EKAM is the product of the moment arm and the ground reaction force (GRF), with the moment arm being a perpendicular distance from the center of rotation in the knee’s coronal plane to the line of action of the GRF vector. Therefore, an intervention strategy that shortens the moment arm by either moving the GRF vector laterally toward the knee center or moving the knee center medially toward the GRF vector can reduce the EKAM.

Lateral wedge insoles are conventionally used to reduce the EKAM in patients with medial knee OA. Moving the GRF vector closer to the knee by shifting the center of pressure (COP) laterally using a lateral wedge insole has been reported to be effective in reducing the EKAM by shortening the moment arm [6,7,8]. Various degrees of wedging are used in the insole, and the wedge is implemented either in only the heel area (hindfoot wedging) or throughout the entire insole (entire wedging). Most studies have been conducted using relatively low wedge angles of 4–6 degrees [9]. However, the effectiveness of lateral wedge insoles on medial knee OA is debatable. Recent meta-analyses and systematic reviews suggest that lateral wedge insoles, regardless of the degree of wedging, have a minimal effect on reducing the EKAM, thus being ineffective for individuals with medial knee OA [9,10,11].

Movements of the proximal and distal segments of a joint are coupled by external moments, closed kinetic chain activities, during weight bearing. Since the subtalar joint’s axis is inclined approximately 42 degrees from the transverse plane and medially deviated about 23 degrees from the foot’s midline [12], triplanar movement in the coronal, sagittal, and transverse planes occurs at the subtalar joint. In closed kinetic chain activities, triplanar movement of the proximal segment of the subtalar joint occurs coupled with that of the distal segment. For instance, when the calcaneus, the subtalar joint’s distal segment, everts in the coronal plane, the proximal segment of the subtalar joint, i.e., the talus and tibia, rotates internally in the transverse plane. Unlike other joints in the human body, the proximal and distal segments of the subtalar joint are connected to each other. The talus, a proximal segment of the subtalar joint, articulates proximally with the tibia (lower leg) and distally with the navicular bone, forming the medial column of the foot along with the cuneiform, and first to third metatarsals. The calcaneus, a distal segment of the subtalar joint, articulates distally with the lateral column of the foot, consisting of the calcaneus, cuboid, and fourth and fifth metatarsals. The lateral and medial columns are articulated with each other. As a result, the proximal and distal segments of the subtalar joint are connected rather than separated from each other. The medial and lateral columns form the medial and lateral longitudinal arches of the foot, respectively, and play an important role in absorbing GRF energy and converting it into propulsion energy during walking [13].

Forefoot eversion affects the movement of both the proximal segment and the distal segment of the subtalar joint through the medial column and lateral column of the foot. In addition, most biomechanical studies on lateral wedge insoles have focused on the lateral shift of the COP, not the medial displacement of the knee. Although there have been kinetic and kinematic studies on the effect of the wedge angle and wedge length on the EKAM, to the best of our knowledge, no study has been conducted on how each hind- and fore-foot eversion causes positional and rotational displacement of the knee.

The purpose of this study is to investigate the effect of hind- and fore-foot eversion on medial displacement of the knee and 3-DoF (degrees of freedom) rotations of the lower leg in a standing posture. Additionally, the present study aims to obtain data for an insole for the knee by assessing foot discomfort and plantar pressure according to each inclination angle of the hindfoot and forefoot.

## 2. Methods

The study protocol was approved and reviewed by the Public Institutional Review Board designated by the Ministry of Health and Welfare, Republic of Korea (Registry No. P01-202211-01-018, approved on 10 November 2022). Informed consent was obtained from all volunteers according to the Declaration of Helsinki. This trial was registered in South Korea’s CRIS database; Clinical trial number: KCT0008189.

### 2.1. Adjustable Foot Eversion-Inversion Platform

**Figure 1:**
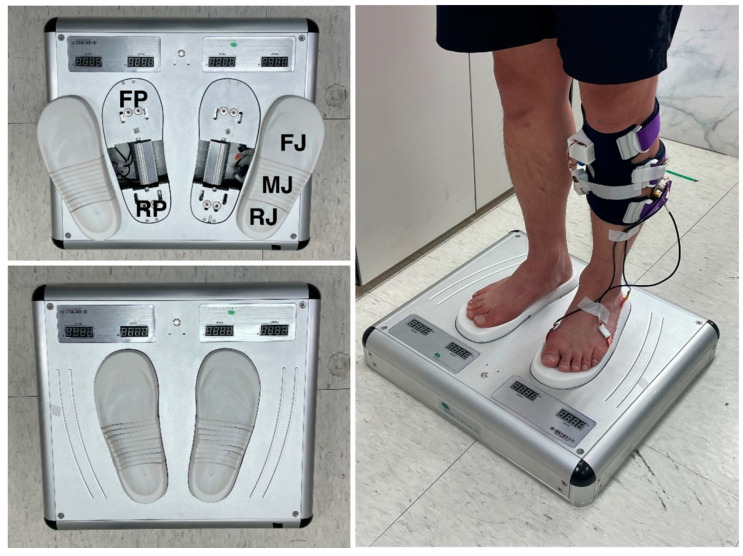
Adjustable Foot Eversion-Inversion Platform (Device for Research, 2021, Biomechanics Research and Development Center, Rhin Rehabilitation Hospital, Republic of Korea); RP, rear plate; FP, front plate; RJ, rear portion of the sole jig; FJ, front portion of the sole jig; MJ, middle portion of the sole jig.

The Adjustable Foot Eversion-Inversion Platform was developed to evert the hindfoot and forefoot individually for pronation of the subtalar joint (Figure 1). The body weight is loaded onto the ground through the calcaneus and heads of metatarsal bones, and the foot arches between the calcaneus and metatarsal heads allow efficient shock absorption and act as a solid lever for propulsion during walking and running. Thus, the platform has a rear plate (RP) and a front plate (FP) placed separately under the hindfoot and forefoot for inclining the calcaneus and the metatarsal heads in the coronal plane, respectively. The front plate extends from just proximal to the metatarsal heads to the front end of the foot, and there is no artificially inclining plate under the foot arches. The sole jig is installed on the rear and front plate, and various sizes are provided at intervals of 5mm in length so it can be replaced and installed on the rear and front plate according to the size of the foot. The sole jig also has a rear (RJ) and front portion (FJ) that are installed on the rear and front plate, and a middle portion (MJ) between the rear and front portion is located under the foot arches. The upper surface of the sole jig has a three-dimensional curved shape corresponding to the plantar surface for maximizing the contact area with the foot sole. In order to not limit the mobility of the medial longitudinal arch of the foot, the jig upper surface was not subjected to weight bearing. To ensure that the mobility of the medial longitudinal arch of the foot is not limited or restricted, the upper surface of the jig beneath it was designed to be either non-weight bearing or minimally weight bearing. By using motors to individually incline the rear and front plates in the coronal plane, the hindfoot and forefoot can be artificially inclined separately. The inclining angles of the rear and front plates were wirelessly controlled. The middle portion of the sole jig (MJ) under the foot arches is sliced in the direction of the foot width and consists of several pieces, and each sliced piece is passively inclined according to the change in the plantar surface. The toe-out angle, defined as the angle between the line from the calcaneus to the second metatarsal bone of both feet and the line between the centers of both hind feet, was 9 degrees and 209 mm, respectively, in the standing position on the platform.

### 2.2. Participants and Procedure

Healthy volunteers were recruited from the local population of the Suwon and Yongin metropolitan areas (Korea) via social media. All participants were required to be over 20 years of age and in good health, without pain in the lower limbs and without musculoskeletal or neurological disorders. Exclusion criteria encompassed foot deformities such as flat foot and cavus foot, a history of spinal or lower limb surgery, asymmetry of body alignment such as scoliosis, discrepancies in hip height, and discrepancies in shoulder height. The study received approval from the Korea National Institutional Review Board, and all participants provided written informed consent.

Prior to the intervention and outcome measurements, participants were instructed to sit in a chair. The calcaneal tuberosity, the first metatarsal head, and the fifth metatarsal head of the dominant foot were palpated, and three film pressure sensors were positioned under each location, fixed with a urethane adhesive film. Subsequently, a leg sleeve equipped with an IMU sensor was palced on the dominant lower leg. After donning the sleeve, participants were asked to assume a standing posture with both feet on the Adjustable Foot Eversion-Inversion Platform. In a comfortable standing posture, markers (diameter 5 mm) were affixed to the center of the patella of both knees, and the standing height and knee height, defined as the vertical distance from the center of the rear jig portion to the marker, were measured using a digital height gauge. A digital camera was installed and secured using a tripod 50 cm in front of the participant’s knee, and a 30 cm-long narrow ruler was positioned vertically as a reference marker between both knees in the coronal plane, where the markers on both patellae were located.

Following all preparations, the rear plate was inclined to elevate the lateral side of the hindfoot in the coronal plane. The rear plate was inclined to 10 degrees in 2-degree increments, commencing from a neutral position with both the rear and front plates at 0 degrees. At each inclination angle of the rear plate, the foreplate was inclined at 2-degree intervals from 0 degrees to the rear-plate inclination angle. At each inclination angle of the rear and front plates, the distance between both knees, tibia rotations, plantar pressure, and foot discomfort were measured.

### 2.3. Measurement of the Distance between Both Knees and Estimation of the Moment Arm

**Figure 2:**
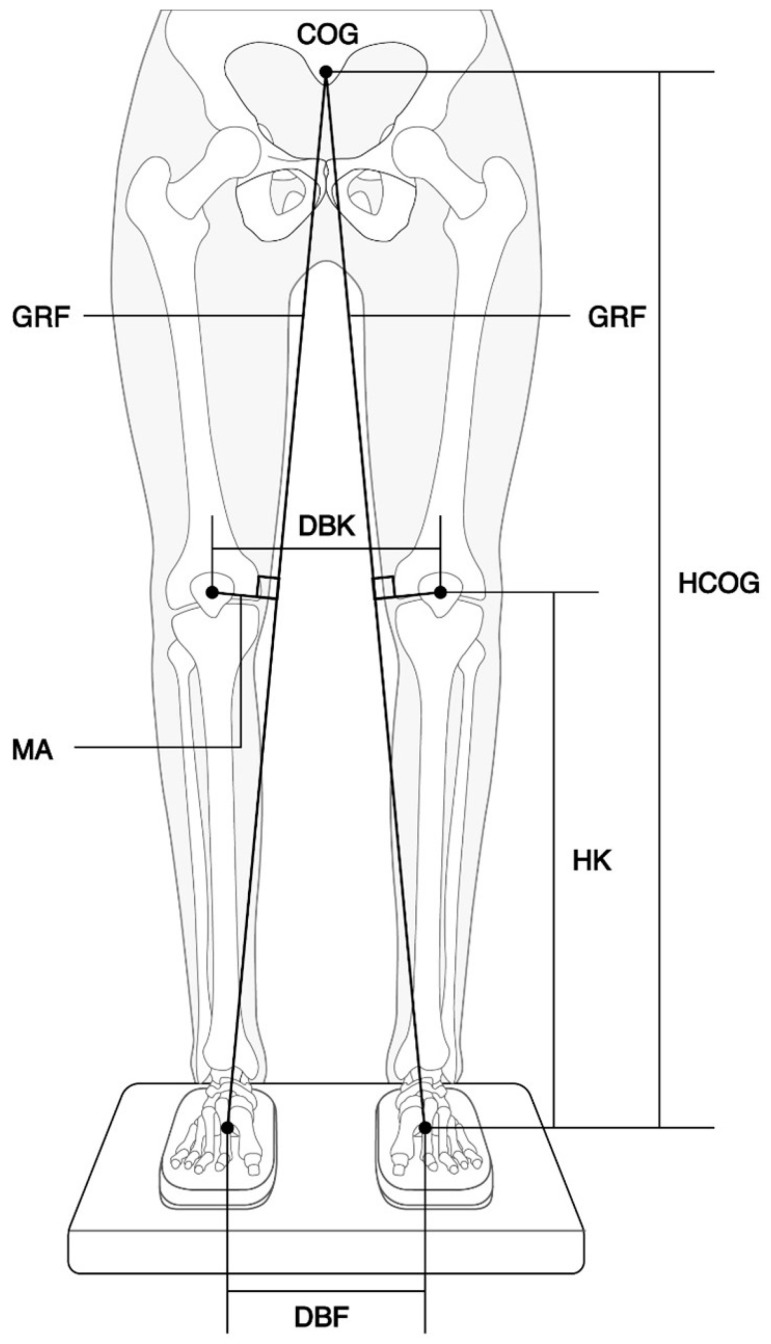
Estimation of the moment arm of the EKAM. MA, moment arm; GRF, ground reaction force; COG, center of gravity; HCOG, height of the COG; HK, height of the knee; DBK, distance between both knees; DBF, distance between the centers of both hind feet.

A blinded investigator measured the distance between both knees using digital images taken at each inclination angle of the rear and front plates. Quantitative analysis of the distance was conducted using image analysis software ((ImageJ, National Institutes of Health, Bethesda, MD, USA, v1.46, https://imagej.net). The moment arm (MA) of the EKAM was calculated using the following equation:

$$MA=\frac{a\times (d-(\left(a-c\right)\times b)/a)}{\sqrt{a^{2}+b^{2}}}$$

where *MA* represents the moment arm, *a* is the height of the COG (HCOG), b is half the distance between the centers of both hind feet (DBF), c is the height of the knee (HK), and *d* is half the distance between both knees (DBK). Detailed information can be found in Figure 2. Since the center of gravity (COG) of a human body in quiet standing is generally accepted to be located at a point equivalent to 57% and 55% of the standing height for males and females, respectively [14]. the height of the COG was estimated by applying these values.

At each inclination angle of the rear and front plates, the decrease in the moment arm and percentage decrease in the EKAM were calculated by the formula given below, respectively:

Decrease in the moment arm = N − I(1)

Percentage decrease in the EKAM = [(N − I)/N] × 100(2)

where N and I are the moment arms at the neutral position (both the rear and front plates at 0 degrees) and at the inclined position, respectively. The decrease in the moment arm is the difference in the length of the moment arm between the neutral position and the inclined position, and a positive value means the knee is medially displaced. The EKAM is the product of the moment arm and the GRF. Given that the magnitude of the GRF remains constant irrespective of the hindfoot and forefoot inclination angles, the percentage decrease in the EKAM’s moment arm and that of the EKAM are equivalent.

### 2.4. Measurement of 3-DoF Rotations of Tibia Using IMU Sensor

A leg sleeve equipped with a 9-axis IMU sensor (Xsens Technologies B.V., 512-MTI-630, Enschede, Netherlands) was worn on the dominant leg to evaluate the 3-DoF rotational displacement of the tibia caused by eversion of the hindfoot and forefoot. The IMU sensor was placed on the medial aspect of the tibial tuberosity, where there is no soft tissue between the tibia and skin, and a silicone film was attached to the bottom of the sensor to eliminate slippage with the skin, thereby minimizing motion artifacts. The 3-DoF tilt signals from the IMU sensor were wirelessly transmitted at a sampling rate of 80–100 Hz to a receiving station connected to a laptop computer. Medial and lateral tilting refer to the tibia’s tilt in the coronal plane (roll), with the angle increasing as the tibia tilts medially. Anterior and posterior tilting involve the tibia’s tilt in the sagittal plane (pitch), with the angle increasing as the tibia tilts posteriorly. Internal and external rotation describe the tibia’s rotation in the transverse plane (yaw), with the angle increasing as the tibia rotates internally.

### 2.5. Assessment of Plantar Pressure and Foot Discomfort

Film pressure sensors (SingleTact, CS15-450N, USA) were employed to assess the changes in pressure applied to the foot sole due to eversion of the hindfoot and forefoot. Since body weight is primarily loaded onto the ground through the calcaneus, the head of the first metatarsal, and the head of the fifth metatarsal, three pressure film sensors were attached under each respective location to measure medial forefoot pressure, lateral forefoot pressure, and heel pressure. The signals from the film pressure sensors were wirelessly transmitted at a sampling rate of 80–100 Hz to a receiving station connected to a laptop computer. The plantar pressure at each inclination condition was compared with the plantar pressure measured in a neutral position with both the rear and front plates at 0 degrees. All signals from the IMU sensor and film pressure sensors were synchronized in time.

In each inclination condition, participants were asked to rate foot discomfort using a 101-point numerical rating scale (NRS) of 0–100, where 0 = no discomfort and 100 = worst discomfort.

### 2.6. Statistical Analysis

In the present study, an intention-to-treat (ITT) approach was employed for data analysis, including all participants in the final analysis. Two-way repeated measures ANOVAs were conducted to determine if changes in outcomes, such as moment arm, knee tilt angles, plantar pressure, and foot discomfort, resulted from the interaction between the “hindfoot eversion angle” and the “forefoot eversion status (i.e., hindfoot-only or entire foot eversion)”. In cases of significant interaction effects, post-hoc pairwise comparisons with Bonferroni correction (α = p/7 = 0.007) were applied. Additionally, at each hindfoot inclination angle, a one-way repeated measures ANOVA with Bonferroni correction was performed to assess the influence of forefoot inclination on dependent variables. Homogeneity of variance was evaluated using Mauchly’s test of sphericity. If homogeneity of variance was violated, Greenhouse–Geisser corrections were applied. All statistical analyses were conducted using Python (version 3.10.9) with the Statsmodels (version 0.14.0) and SciPy (version 1.9.0) packages (Python Software Foundation, Wilmington, DE, USA).

## 3. Results

A total of 29 healthy volunteers were enrolled in this study. However, two volunteers with flat feet and one volunteer with mild scoliosis were excluded. Consequently, 26 participants completed the study and were included in the analysis. Table 1 presents the anthropometric data of the participants, while Table 2 displays the descriptive statistics of the outcomes by the inclination angles of the hindfoot and forefoot. Significant interactions between hindfoot and forefoot eversion were found for all dependent variables, leading to post-hoc pairwise comparisons with Bonferroni correction (α = p/7 = 0.007). Due to violations of sphericity assumptions (Mauchly’s tests, all *p* < 0.05), Greenhouse–Geisser corrections were applied in the analysis.

### 3.1. Effect of the Hind- and Fore-Foot Eversion on Knee Displacement in the Coronal Plane

**Figure 3:**
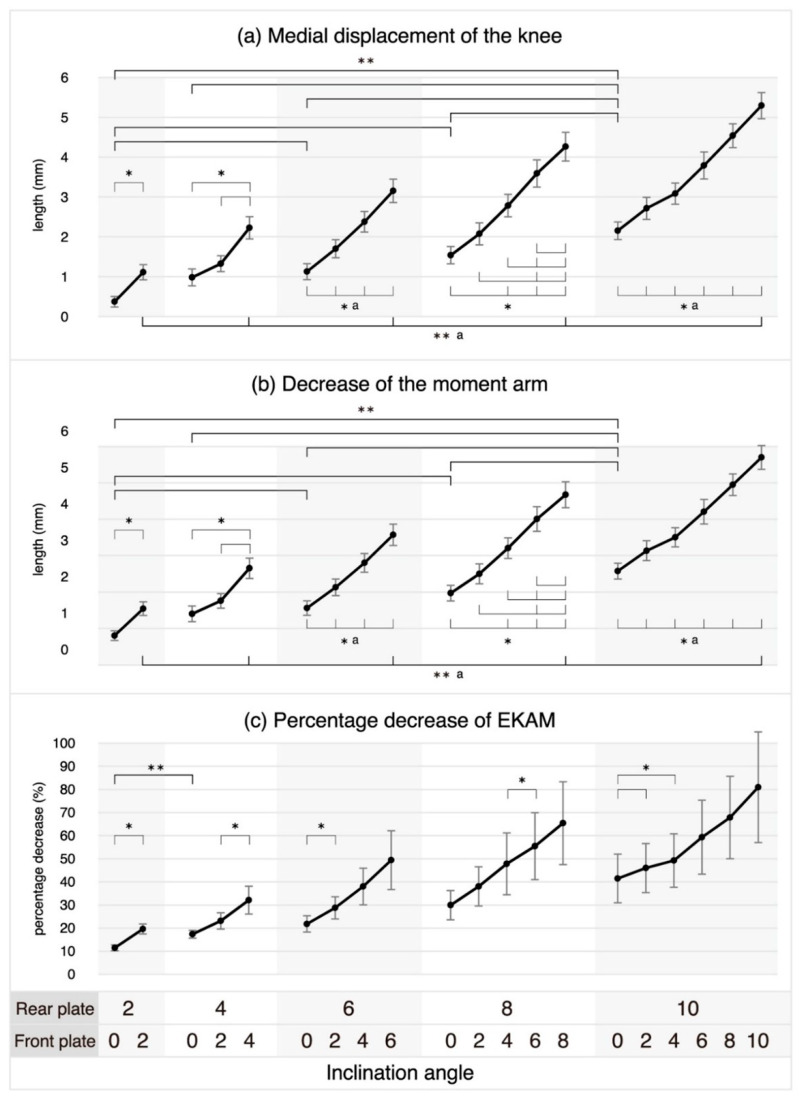
Positional displacement of the knee by inclining the hind- and fore-foot. (**a**) Medial displacement of the knee is half the difference in the DBK (distance between both knees) measured between the neutral position (both the rear and front plates at 0 degrees) and the inclination position, where a positive value indicates that the knee is displaced towards the midline of the body (medially) at the inclination position. (**b**) The decrease in the moment arm is the difference in the moment arm measured between the neutral position and the inclination position, where a positive value indicates that the moment arm measured at the inclination position is shorter than that measured at the neutral position. (**c**) See Methods for a description of percentage decrease in the EKAM. Bars show the mean ± standard error. * Statistical significance was determined at *p* ≤ 0.05 in paired *t*-tests for the rear plate’s inclination angle at 2°, and in one-way repeated measures ANOVAs, it was established at *p* ≤ 0.017 for 4°, *p* ≤ 0.008 for 6°, *p* ≤ 0.005 for 8°, and *p* ≤ 0.003 for 10°. ** Statistical significance is indicated by bold line and was established at *p* ≤ 0.0072 for post-hoc pairwise comparisons, which were performed after identifying significant interactions in two-way repeated measures ANOVAs. ^a^ Indicates statistically significant results in all comparisons.

In hindfoot-only eversion, no statistically significant differences were observed in the medial displacement of the knee or between decreases in the moment arm eversion angles of 2° vs. 4°, 4° vs. 6°, 4° vs. 8°, and 6° vs. 8°. However, for the other eversion angle comparisons in hindfoot-only eversion, as the eversion angle increased, the knee was significantly displaced medially, and the length of the moment arm also decreased significantly. In entire-foot eversion, where the forefoot was everted as much as the hindfoot eversion angle, as the eversion angle increased, the knee was significantly displaced medially, and the length of the moment arm also significantly decreased. Table 3 presents the results of the post-hoc pairwise comparisons following the identification of significant interactions in two-way repeated measures ANOVA. At each hindfoot eversion angle, as the forefoot eversion increased, the knee was significantly displaced medially and the moment arm decreased significantly, except when the forefoot eversion increased from 0° to 2° at the hindfoot eversion angle of 4°, from 0° to 2°, and from 2° to 4° at the hindfoot eversion angle of 8° (Figure 3).

Except for the comparison between 2° and 4° hindfoot eversion angles, no statistically significant differences were observed in the percentage decrease in the EKAM in hindfoot-only eversion at any other angle comparisons. In entire-foot eversion, although a percentage decrease in the EKAM was observed as the eversion angle increased, none of the changes were statistically significant (Table 3). At each hindfoot eversion angle, the percentage decrease in the EKAM due to increasing forefoot eversion was found to be statistically significant for specific combinations, as shown in Table 3 and Figure 3. However, the percentage decrease was not found to be statistically significant for any other combinations.

### 3.2. Effect of the Hind- and Fore-Foot Eversion on Lower Leg Rotation

**Figure 4:**
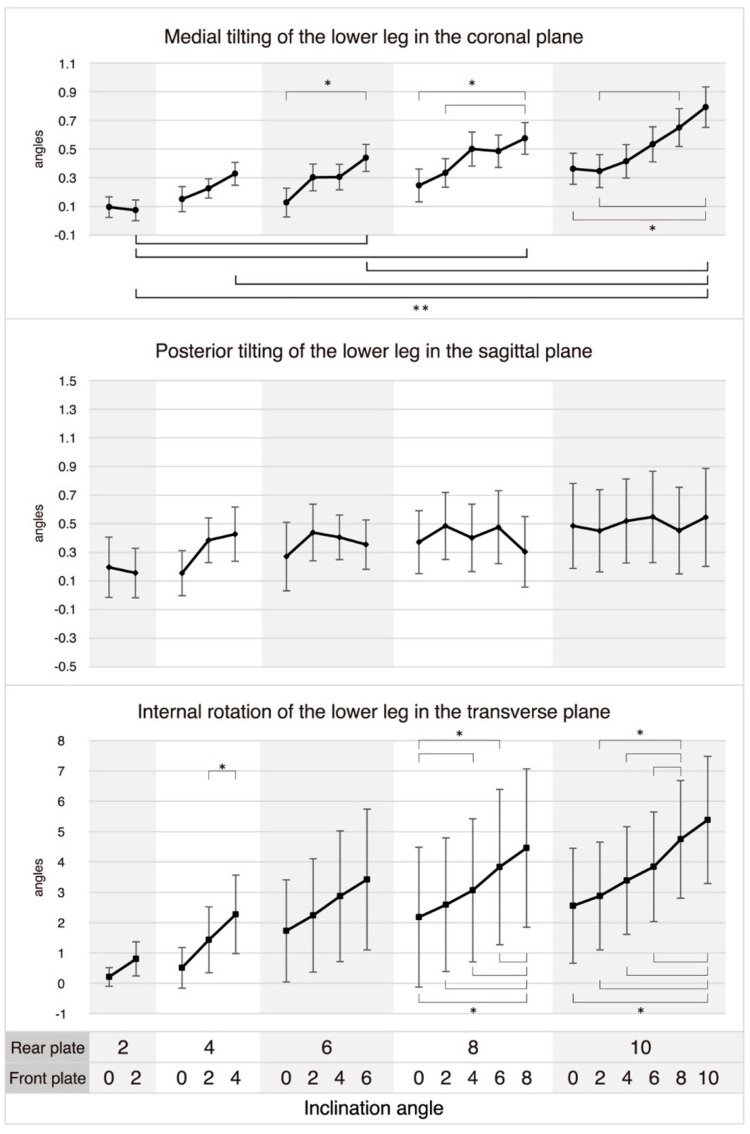
Triplanar rotation of the tibia by inclining the hind− and fore−foot. Medial tilting, posterior tilting, and internal rotation of the lower leg are the differences in roll, pitch, and yaw values obtained from the IMU sensor between the neutral position and the inclination position, where positive values indicate that the lower leg is more medially tilted, posteriorly tilted, and internally rotated at the inclined position compared to the neutral position in the coronal, sagittal, and transverse planes, respectively. Bars show the mean ± standard error. * Statistical significance was determined at *p* ≤ 0.05 in paired *t*-tests for the rear plate’s inclination angle at 2°, and in one-way repeated measures ANOVAs, it was established at *p* ≤ 0.017 for 4°, *p* ≤ 0.008 for 6°, *p* ≤ 0.005 for 8°, and *p* ≤ 0.003 for 10°. ** Statistical significance is indicated by bold line and was established at *p* ≤ 0.0072 for post-hoc pairwise comparisons, which were performed after identifying significant interactions in two-way repeated measures ANOVAs.

In hindfoot-only eversion, increasing the eversion angle did not significantly change the tilting and rotation of the lower leg in the coronal, sagittal, or transverse planes. In contrast, in entire-foot eversion, as the eversion angle increased from 2° to 6°, 8°, and 10°, from 4° to 10°, and from 6° to 10°, the lower leg showed statistically significant medial tilting in the coronal plane (Ps ≤ 0.072), but there were no significant changes in the lower leg’s tilt or rotation angle in the sagittal and transverse planes as the entire foot’s eversion angle increased. At each hindfoot eversion angle, the lower leg showed statistically significant medial tilting with a forefoot eversion increase from 0° to 6° at hindfoot eversion angles of 6°; from 0° to 8°, and from 2° to 8° at 8°; from 0° to 10°, from 2° to 8°, and from 2° to 10° at 10°. Furthermore, the lower leg showed statistically significant internal rotation in the transverse plane with a forefoot eversion increase from 2° to 4° at a hindfoot eversion angle of 4°; from 0° to 4°, from 0° to 6°, from 0° to 8°, from 2° to 8°, from 4° to 8°, and from 6° to 8° at 8°; from 0° to 10°, from 2° to 8°, from 2° to 10°, from 4° to 8°, from 4° to 10°, from 6° to 8°, and from 6° to 10° at 10°. In contrast, forefoot eversion at each hindfoot eversion angle did not cause any significant changes in the lower leg’s tilt angle in the sagittal plane (Figure 4).

### 3.3. Effect of the Hind- and Fore-Foot Eversion on Plantar Pressure and Foot Discomfort

**Figure 5:**
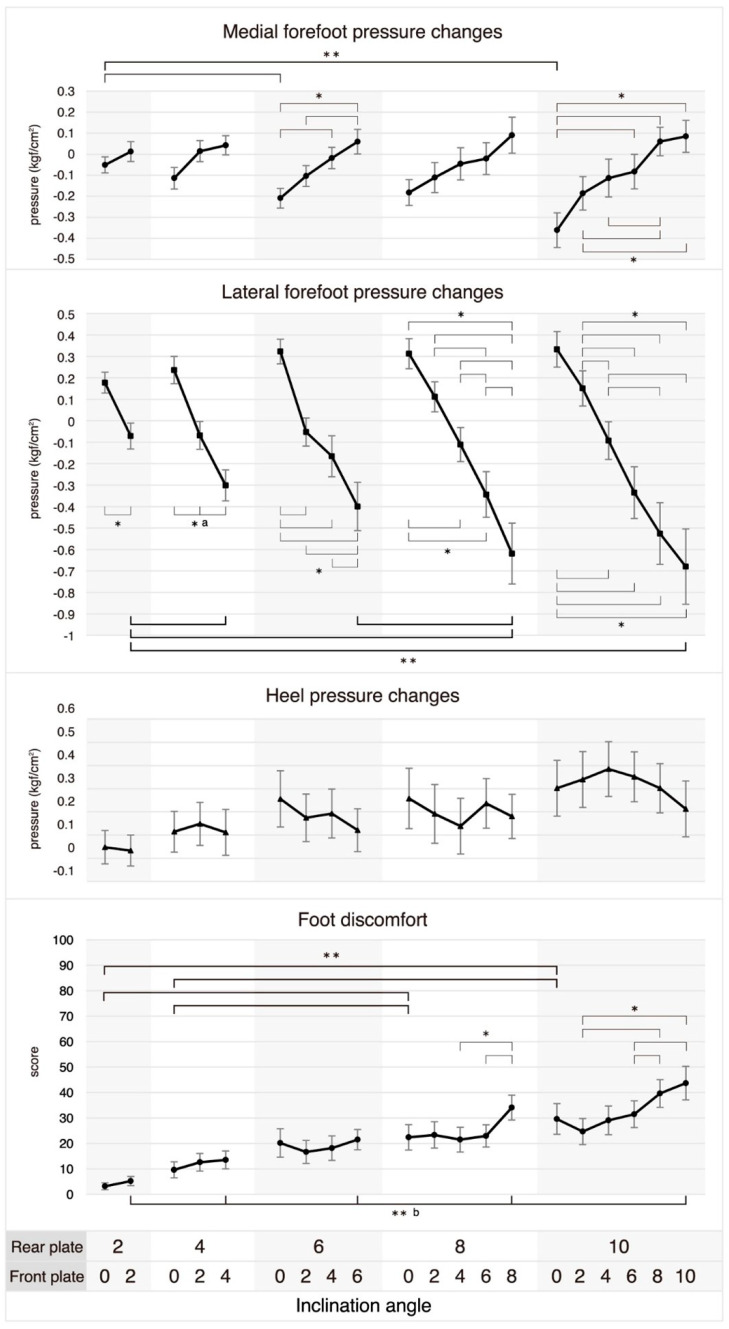
Plantar pressure changes and foot discomfort by inclining the hind−and fore−foot. Plantar pressure change is the difference in plantar pressure measured between the neutral position with both the rear and front plates at 0 degrees and the inclination position, where a negative value indicates an increase in pressure. Bars show the mean ± standard error. * Statistical significance was determined at *p* ≤ 0.05 in paired *t*-tests for the rear plate’s inclination angle at 2°, and in one-way repeated measures ANOVAs, it was established at *p* ≤ 0.017 for 4°, *p* ≤ 0.008 for 6°, *p* ≤ 0.005 for 8°, and *p* ≤ 0.003 for 10°. ** Statistical significance is indicated by bold line and was established at *p* ≤ 0.0072 for post-hoc pairwise comparisons, which were performed after identifying significant interactions in two-way repeated measures ANOVAs. ^a^ Indicates statistically significant results in all comparisons. ^b^ Indicates statistically significant results in all comparisons except between 2° and 4°, and between 8° and 10°.

In hindfoot-only eversion, as the eversion angle increased from 2° to 6° and 10°, the medial forefoot pressure significantly decreased (*p* = 0.003 and *p* < 0.001). Conversely, in entire-foot eversion, as the eversion angle increased from 2° to 4°, 8°, and 10°, and from 6° to 8°, the lateral forefoot pressure significantly increased (Ps = 0.006, 0.002, 0.006, and 0.004). However, in both hindfoot-only eversion and entire-foot eversion, no significant changes in heel pressure were observed as the eversion angle increased. At the hindfoot eversion angle of 6°, the medial forefoot pressure significantly decreased as the forefoot eversion angle increased from 0° to 4° and 6°, and from 2° to 6° (Ps = 0.006, 0.003, and 0.008). In contrast, except when the forefoot eversion angle increased from 2° to 4° at the hindfoot eversion angle of 6° (*p* = 0.659), from 0° to 2° and from 2° to 4° at the hindfoot eversion angle of 8° (*p* = 0.020 and *p* = 0.006), and from 0° to 2°, from 4° to 6°, from 6° to 8° and 10°, and from 8° to 10° at the hindfoot eversion angle of 10° (Ps = 0.011, 0.016, 0.046, 0.028, and 0.279), the lateral forefoot pressure significantly increased in all cases as the forefoot eversion angle increased at each hindfoot eversion angle. However, no significant changes in heel pressure were observed as the forefoot eversion angle increased at each eversion hindfoot angle (Figure 5).

Foot discomfort significantly increased in hindfoot-only eversion when the eversion angle increased from 2° to 8° and 10° (both *p* < 0.001), and from 4° to 8° and 10° (*p* = 0.009 and *p* = 0.004). In the case of entire-foot eversion, foot discomfort significantly increased in all cases with an increase in eversion angle, except when the angle increased from 2° to 4° and from 8° to 10° (*p* = 0.075 and *p* = 0.047). Foot discomfort significantly increased at an eversion angle of the hindfoot of 8° when the forefoot eversion angle increased from 4° to 8° and from 6° to 8° (*p* = 0.002 and *p* = 0.001). At an eversion angle of the hindfoot of 10°, foot discomfort significantly increased when the forefoot eversion angle increased from 2° to 8° and 10° (*p* < 0.001 and *p* = 0.003), and from 6° to 8° and 10° (*p* < 0.001 and *p* = 0.002) (Figure 5).

## 4. Discussion

Although the present study has several limitations, such as its focus on healthy adults and assessment in standing posture, it reveals novel findings not previously reported. As the hindfoot eversion angle increases, the moment arm of the EKAM predictably decreases. Similarly, as the forefoot everts more within the range of the hindfoot eversion angle, the moment arm of the EKAM becomes shorter. However, the more the hindfoot everts, and the more the forefoot everts toward the hindfoot eversion angle, the greater the pressure on the lateral forefoot and the more the foot discomfort increases. According to the findings of this study, it is worth considering incorporating hindfoot eversion angles ranging from 6 to 10 degrees and forefoot eversion angles from approximately 40% to 60% of the hindfoot into the biomechanical design of insoles for knee OA patients.

This study may suggest that subtalar pronation caused by foot eversion leads to the shortening of the moment arm by moving the knee center medially toward the GRF vector via internal rotation and medial tilting of the lower leg. The hip joint and femur’s unique anatomy may contribute to this motion. The hip allows 3-DoF rotational movement, and the femur’s lateral angulation can cause medial knee displacement during internal rotation. This supports previous findings of reduced EKAM during toe-in gait [15]. Further studies on subtalar pronation and hip-joint rotations during weight-bearing activities are needed to expand on this research. For evaluating the effect of midfoot height on knee OA, the lower navicular height in patients with medial knee OA may relate to the alleviation of knee symptoms, and these results may be another supportive clue for our results [16]. Increased hindfoot eversion and forefoot inversion are associated with reduced knee adduction moments during the stance phase of gait, suggesting that medial knee joint loading is reduced in people with OA who walk with greater foot pronation [17]. Taken together with our results, the modification of the hindfoot and forefoot angles would modify the EKAM, and may present merit for reducing burdens in patients with medial knee OA.

Unlike other human joints, the proximal and distal segments of the subtalar joint are interconnected. The calcaneus, the distal segment of the subtalar joint, forms the lateral column by connecting to the cuboid bone and the lateral two rays of the metatarsal bone. The talus, the proximal segment of the subtalar joint, forms the medial column by connecting to the navicular bone, the cuneiforms, and the medial three rays of the metatarsal bone. As a result, the medial column of the foot is connected to the talus, the subtalar joint’s proximal segment, while the lateral column is connected to the subtalar joint’s distal segment. Additionally, the height of the medial column is greater than that of the lateral column. Therefore, when both the hindfoot and forefoot evert during weight-bearing activities, a separating force may occur between the medial and lateral columns, potentially inducing foot pain. If the forefoot eversion angle is smaller relative to the hindfoot eversion angle, there is less medial tilting of the lower leg and a smaller reduction in the moment arm. This may be due to the connection between the medial and lateral columns of the foot, resulting in less subtalar pronation.

There were three limitations noted in our study. The first one was that it only evaluated the standing posture in healthy adults. Therefore, further biomechanical studies and clinical randomized controlled trials are necessary to investigate the effects of insoles incorporating the proposed eversion angles during various weight-bearing activities in patients with knee OA. In addition, although substantial percentage decreases in the EKAM were observed in this study, they were not statistically significant. This lack of significance may be attributed to a type II error resulting from a large standard error, which could be due to increased variability in the percentage change. Therefore, further research with a larger sample size is needed to reduce the standard error and improve the statistical power. The second was that, while our platform may be useful for studying the effect of foot eversion on knee displacement, it may not accurately replicate the natural movement of the foot during weight-bearing activities. Thus, our findings were most representative of the human foot and ankle motion on the knee; however, these results were also a little different from natural dynamic human motion. Lastly, our study only assesses foot discomfort and plantar pressure according to each inclination angle of the hindfoot and forefoot, which may not fully capture the complexity of foot mechanics.

## 5. Conclusions

In this study, when the hindfoot eversion angle increases, the moment arm of the EKAM predictably decreases in healthy subjects. Similarly, as the forefoot everts more within the range of the hindfoot eversion angle, the moment arm of the EKAM becomes shorter. However, the more the hindfoot everts, and the more the forefoot everts toward the hindfoot eversion angle, the greater the pressure on the lateral forefoot and the more the foot discomfort increases. Our findings have merit in furthering understanding of the biomechanical role of insoles for knee OA patients.

## Figures and Tables

**Table 1:** Anthropometric characteristics of the participants.

	Mean ± Standard Deviation (Min, Max)
Age (years)	40.5 ± 12.6 (25, 68)
Gender (male:female)	13:13
Height (cm)	167 ± 7.66 (153, 181)
Weight (kg)	64.5 ± 16.13 (43, 110)
Body mass index (kg/cm^2^)	22.92 ± 4.36 (18.4, 33.6)

**Table 2:** Descriptive statistics of the outcomes by the inclination angles of the hind- and fore-foot.

Inclination Angle		Positional Displacement					Rotational Displacement			Plantar Pressure Change			Foot Discomfort
Rear Plate	Front Plate	DBK	MA	MD	DM	PD	MT	PT	IR	MP	LP	HP	
2	0	142.17 ± 25.01	16.59 ± 12.25	0.37 ± 0.69	0.36 ± 0.68	2.69 ± 6.63	0.10 ± 0.37	0.20 ± 1.10	0.21 ± 1.48	−0.05 ± 0.19	0.18 ± 0.25	0.05 ± 0.37	3.15 ± 6.95
	2	140.68 ± 24.90	15.85 ± 12.18	1.11 ± 0.98	1.10 ± 0.98	10.84 ± 11.19	0.07 ± 0.39	0.16 ± 0.90	0.80 ± 2.70	0.01 ± 0.24	−0.07 ± 0.31	0.03 ± 0.34	5.19 ± 9.35
4	0	140.94 ± 24.86	16.00 ± 12.21	0.98 ± 1.11	0.96 ± 1.11	8.57 ± 8.80	0.15 ± 0.45	0.15 ± 0.82	0.51 ± 3.22	−0.11 ± 0.26	0.24 ± 0.32	0.11 ± 0.45	9.63 ± 16.35
	2	140.25 ± 24.87	15.64 ± 12.20	1.33 ± 1.04	1.32 ± 1.03	14.34 ± 18.35	0.23 ± 0.35	0.38 ± 0.81	1.43 ± 5.20	0.01 ± 0.25	−0.07 ± 0.33	0.15 ± 0.47	12.59 ± 17.94
	4	138.45 ± 24.29	14.74 ± 11.90	2.23 ± 1.46	2.21 ± 1.45	23.31 ± 31.23	0.33 ± 0.41	0.43 ± 0.98	2.27 ± 6.22	0.04 ± 0.23	−0.30 ± 0.37	0.11 ± 0.50	13.52 ± 18.12
6	0	140.65 ± 25.12	15.84 ± 12.30	1.13 ± 1.04	1.12 ± 1.04	13.03 ± 18.35	0.13 ± 0.52	0.27 ± 1.24	1.73 ± 8.08	−0.21 ± 0.24	0.32 ± 0.29	0.26 ± 0.62	20.19 ± 29.14
	2	139.50 ± 25.19	15.27 ± 12.33	1.70 ± 1.18	1.69 ± 1.18	19.99 ± 24.95	0.30 ± 0.49	0.44 ± 1.03	2.24 ± 8.96	−0.10 ± 0.25	−0.05 ± 0.33	0.17 ± 0.52	16.67 ± 23.45
	4	138.15 ± 24.87	14.59 ± 12.19	2.38 ± 1.35	2.36 ± 1.34	29.22 ± 41.18	0.30 ± 0.47	0.40 ± 0.81	2.87 ± 10.34	−0.02 ± 0.26	−0.17 ± 0.49	0.19 ± 0.54	18.15 ± 25.04
	6	136.59 ± 24.81	13.82 ± 12.17	3.15 ± 1.53	3.13 ± 1.52	40.63 ± 66.02	0.44 ± 0.49	0.35 ± 0.89	3.42 ± 11.13	0.06 ± 0.30	−0.40 ± 0.57	0.12 ± 0.47	21.48 ± 20.75
8	0	139.82 ± 25.43	15.43 ± 12.46	1.54 ± 1.12	1.53 ± 1.12	21.13 ± 32.91	0.25 ± 0.60	0.37 ± 1.14	2.18 ± 11.06	−0.18 ± 0.31	0.31 ± 0.36	0.26 ± 0.66	22.41 ± 25.96
	2	138.75 ± 25.75	14.90 ± 12.63	2.07 ± 1.43	2.06 ± 1.42	29.26 ± 44.03	0.33 ± 0.52	0.48 ± 1.21	2.59 ± 10.57	−0.11 ± 0.36	0.11 ± 0.36	0.19 ± 0.65	23.33 ± 26.85
	4	137.33 ± 25.24	14.19 ± 12.35	2.79 ± 1.47	2.77 ± 1.47	39.02 ± 69.49	0.50 ± 0.62	0.40 ± 1.22	3.06 ± 11.32	−0.05 ± 0.39	−0.11 ± 0.40	0.14 ± 0.61	21.48 ± 25.26
	6	135.72 ± 24.98	13.39 ± 12.24	3.59 ± 1.77	3.57 ± 1.76	46.68 ± 75.10	0.49 ± 0.59	0.48 ± 1.32	3.83 ± 12.28	−0.02 ± 0.39	−0.34 ± 0.54	0.24 ± 0.55	22.96 ± 22.59
	8	134.38 ± 25.35	12.72 ± 12.37	4.26 ± 1.87	4.24 ± 1.86	56.61 ± 93.05	0.57 ± 0.57	0.30 ± 1.28	4.46 ± 12.51	0.09 ± 0.44	−0.62 ± 0.72	0.18 ± 0.49	34.07 ± 25.39
10	0	138.60 ± 25.68	14.82 ± 12.61	2.15 ± 1.14	2.14 ± 1.13	32.70 ± 54.69	0.36 ± 0.56	0.48 ± 1.54	2.56 ± 9.09	−0.36 ± 0.42	0.33 ± 0.42	0.30 ± 0.61	29.63 ± 31.38
	2	137.47 ± 25.75	14.26 ± 12.62	1.71 ± 1.43	2.70 ± 1.42	37.20 ± 55.13	0.35 ± 0.60	0.45 ± 1.49	2.88 ± 8.52	−0.19 ± 0.41	0.15 ± 0.42	0.34 ± 0.61	24.63 ± 26.60
	4	136.73 ± 25.34	13.89 ± 12.43	3.08 ± 1.38	3.06 ± 1.37	40.46 ± 60.02	0.41 ± 0.61	0.52 ± 1.53	3.39 ± 8.52	−0.11 ± 0.46	−0.09 ± 0.45	0.38 ± 0.60	29.07 ± 29.39
	6	135.32 ± 24.90	13.19 ± 12.20	3.79 ± 1.76	3.77 ± 1.75	50.55 ± 83.04	0.53 ± 0.63	0.55 ± 1.66	3.84 ± 8.66	−0.08 ± 0.42	−0.33 ± 0.62	0.35 ± 0.55	31.48 ± 27.31
	8	133.82 ± 24.91	12.45 ± 12.21	4.54 ± 1.56	4.51 ± 1.55	59.04 ± 92.65	0.65 ± 0.68	0.45 ± 1.57	4.75 ± 9.30	0.06 ± 0.35	−0.53 ± 0.73	0.30 ± 0.54	39.63 ± 28.28
	10	132.31 ± 24.84	11.70 ± 12.17	5.29 ± 1.71	5.26 ± 1.70	72.17 ± 124.48	0.79 ± 0.73	0.54 ± 1.78	5.39 ± 10.05	0.08 ± 0.39	−0.68 ± 0.90	0.21 ± 0.61	43.70 ± 34.24

DBK, distance between both knees; MA, moment arm; MD, medial displacement of the knee; DM, decrease of the moment arm; PD, percentage decrease of the EKAM; MT, medial tilting of the tibia in the coronal plane; PT, posterior tilting of the tibia in the sagittal plane; IR, internal rotation of the tibia in the transverse plane; MP, medial forefoot pressure; LP, lateral forefoot pressure; HP, heel pressure. Values are mean ± standard deviation.

**Table 3:** Post-hoc pairwise comparison following identification of significant interactions in two-way repeated measures ANOVAs.

Eversion Angle ^a^		Medial Displacement of the Knee	Decrease of the Moment Arm	Percentage Decrease of the EKAM
Comparison in Hindfoot-Only Eversion				
2/0 vs.	4/0	−0.613 ± 0.174 (0.016)	−0.599 ± 0.175 (0.021)	−5.885 ± 1.423 (0.003) *
	6/0	−0.759 ± 0.159 (<0.001) *	−0.758 ± 0.158 (<0.001) *	−10.341 ± 3.512 (0.067)
	8/0	−1.172 ± 0.178 (<0.001) *	−1.168 ± 0.177 (<0.001) *	−18.439 ± 6.395 (0.078)
	10/0	−1.783 ± 0.206 (<0.001) *	−1.775 ± 0.205 (<0.001) *	−30.010 ± 10.719 (0.095)
4/0 vs.	6/0	−0.147 ± 0.157 (1.000)	−0.159 ± 0.160 (1.000)	−4.456 ± 2.572 (0.950)
	8/0	−0.559 ± 0.190 (0.067)	−0.569 ± 0.190 (0.059)	−12.555 ± 5.582 (0.332)
	10/0	−1.170 ± 0.243 (<0.001) *	−1.176 ± 0.243 (<0.001) *	−24.126 ± 9.866 (0.216)
6/0 vs.	8/0	−0.413 ± 0.118 (0.017)	−0.410 ± 0.117 (0.017)	−8.099 ± 3.335 (0.224)
	10/0	−1.024 ± 0.181 (<0.001) *	−1.017 ± 0.179 (<0.001) *	−19.669 ± 7.678 (0.166)
8/0 vs.	10/0	−0.611 ± 0.113 (<0.001) *	−0.607 ± 0.112 (<0.001) *	−11.571 ± 4.535 (0.170)
Comparison in entire foot eversion				
2/2 vs.	4/4	−1.116 ± 0.168 (<0.001) *	−1.110 ± 0.167 (<0.001) *	−12.468 ± 4.454 (0.095)
	6/6	−2.043 ± 0.235 (<0.001) *	−2.030 ± 0.234 (<0.001) *	−29.789 ± 11.097 (0.125)
	8/8	−3.152 ± 0.255 (<0.001) *	−3.132 ± 0.254 (<0.001) *	−45.770 ± 16.232 (0.091)
	10/10	−4.183 ± 0.240 (<0.001) *	−4.157 ± 0.238 (<0.001) *	−61.325 ± 22.291 (0.107)
4/4 vs.	6/6	−0.927 ± 0.230 (0.004) *	−0.921 ± 0.229 (0.004) *	−17.321 ± 7.388 (0.270)
	8/8	−2.035 ± 0.246 (<0.001) *	−2.022 ± 0.245 (<0.001) *	−33.302 ± 12.381 (0.123)
	10/10	−3.067 ± 0.230 (<0.001) *	−3.047 ± 0.229 (<0.001) *	−48.858 ± 18.428 (0.135)
6/6 vs.	8/8	−1.109 ± 0.224 (<0.001) *	−1.101 ± 0.222 (<0.001) *	−15.981 ± 5.445 (0.069)
	10/10	−2.140 ± 0.189 (<0.001) *	−2.126 ± 0.188 (<0.001) *	−31.536 ± 11.426 (0.104)
8/8 vs.	10/10	−1.031 ± 0.217 (<0.001) *	−1.025 ± 0.216 (<0.001) *	−15.555 ± 6.360 (0.215)
Comparison between hindfoot-only eversion and entire foot eversion				
2/0 vs. 2/2		−0.743 ± 0.751 (<0.001) *	−0.742 ± 0.747 (<0.001) *	−8.156 ± 11.278 (<0.001) *
4/0 vs. 4/4		−1.247 ± 0.838 (<0.001) *	−1.253 ± 0.850 (<0.001) *	−14.739 ± 27.984 (0.011)
6/0 vs. 6/6		−2.027 ± 1.199 (<0.001) *	−2.014 ± 1.193 (<0.001) *	−27.604 ± 52.376 (0.011)
8/0 vs. 8/8		−2.723 ± 1.474 (<0.001) *	−2.706 ± 1.466 (<0.001) *	−35.487 ± 64.368 (0.008)
10/0 vs. 10/10		−3.143 ± 1.336 (<0.001) *	−3.124 ± 1.329 (<0.001) *	−39.471 ± 72.585 (0.009)

Values represent mean differences ± standard error (*p*-value). * Indicates statistically significant results (*p* ≤ 0.0072). ^a^ Values before the slash (/) denote the inclination angle of the rear plate (hindfoot eversion angle), while values after the slash indicate the inclination angle of the front plate (forefoot eversion angle).

## Data Availability

The data presented in this study are available on request from the corresponding author.
